# Human MUS81: A Fence-Sitter in Cancer

**DOI:** 10.3389/fcell.2021.657305

**Published:** 2021-03-15

**Authors:** Sisi Chen, Xinwei Geng, Madiha Zahra Syeda, Zhengming Huang, Chao Zhang, Songmin Ying

**Affiliations:** ^1^International Institutes of Medicine, The Fourth Affiliated Hospital of Zhejiang University School of Medicine, Yiwu, China; ^2^Key Laboratory of Respiratory Disease of Zhejiang Province, Department of Pharmacology and Department of Respiratory and Critical Care Medicine of the Second Affiliated Hospital, Zhejiang University School of Medicine, Hangzhou, China

**Keywords:** human MUS81, endonuclease, DNA damage response, cancer therapy, chromosomal instability

## Abstract

MUS81 complex, exhibiting endonuclease activity on specific DNA structures, plays an influential part in DNA repair. Research has proved that MUS81 is dispensable for embryonic development and cell viability in mammals. However, an intricate picture has emerged from studies in which discrepant gene mutations completely alter the role of MUS81 in human cancers. Here, we review the recent understanding of how MUS81 functions in tumors with distinct genetic backgrounds and discuss the potential therapeutic strategies targeting MUS81 in cancer.

## Introduction

Mus81 was first identified by its cooperation with the homologous recombination (HR) protein Rad54 in yeast (Boddy et al., 2000; Interthal and Heyer, 2000; Haber and Heyer, 2001), indicating its possible role in DNA repair. Owing to its high evolutionary conservation, human MUS81 was subsequently discovered (Chen et al., 2001). The MUS81 protein possesses a characteristic ERCC4 nuclease domain containing the VERKX_3_D motif, which is indispensable for the endonuclease activity (Chen et al., 2001). The abundance of human MUS81 augments unequivocally when cells are exposed to replication stress (Chen et al., 2001). Human MUS81 localizes to damaged DNA sites under replication stress (Gao et al., 2003). MUS81-depleted U2OS cells exhibit elevated level of chromosomal bridges and micronuclei, characteristic features of DNA damage (Ying et al., 2013). MUS81-deficient cells and mice are intolerant to mitomycin C (MMC), an interstrand crosslinking agent, while MUS81 haploinsufficiency results in genomic instability (McPherson et al., 2004). Altogether, mammalian MUS81 is unambiguously a DNA damage repair protein.

DNA damage response (DDR) defects are common occurrences in multiple cancers, manifesting as mutation and inactivation of DDR-relative proteins. In recent findings, it is becoming increasingly evident that MUS81 has close relationships with cancers. Interestingly, MUS81 is helpful for tumor survival in some cases but lethal to them in other cases. For instance, endonuclease activity of MUS81 is crucial for survival of BRCA2-insufficient cancer cells (Lai et al., 2017; Lemaçon et al., 2017). Nevertheless, in WRN-depleted microsatellite instability (MSI) cancer cells, MUS81 complex shatters chromosomes, which causes apoptosis of cancer cells (van Wietmarschen et al., 2020).

Herein, this review focuses on recent understanding of how MUS81 works in distinct cancer cells and discuss why MUS81 causes such different consequences as black and white in tumors.

## Mechanisms of MUS81 in DNA Repair

### Molecular Mechanism of Substrate Recognition and Cleavage by MUS81

Human MUS81 is a substrate selective endonuclease and exhibits a wide range of specificity for replication forks, 3′-flap structures, Holliday junctions (HJs), and D-loops (Chen et al., 2001; Constantinou et al., 2002; Zeng et al., 2009).

The crystal structure of human MUS81–essential meiotic structure-specific endonuclease 1 (MUS81–EME1) combined with 3′ flaps demonstrates the recognition and cleavage mechanism of MUS81 complex (Chang et al., 2008; Gwon et al., 2014). Binding of 3′-flap DNA induces rotation of the helix–hairpin–helix (HhH_2_) heterodimer of the MUS81–EME1 complex. The disordered loop of the EME1 linker becomes ordered, which subsequently unmasks the hydrophobic wedge and forms the 5′ end binding pocket of MUS81 (Gwon et al., 2014). The binding pocket accommodates the 5′ nicked end of the 3′ flap DNA, which is pivotal for the substrate specificity (Tsodikov et al., 2005; Chang et al., 2008). HhH2 of EME1 and HhH2 of MUS81 are able to interact with the pre- and the postnick DNA strands, respectively, as a result of the conformational changes (Gwon et al., 2014). The wedge bends the substrate, which put the 3′ end into the ERCC4 site of MUS81 and assists the substrate cleavage (Enzlin and Schärer, 2002; Gwon et al., 2014).

### MUS81–EME2 Promotes DNA Replication Completion in S Phase

Replication forks are prone to stall when encountering obstacles during DNA replication (Liao et al., 2018). The recovery of the replication forks is necessary for faithful DNA replication and cell division (Bryant et al., 2009). MUS81–EME2 can cleave reversed replication forks (Amangyeld et al., 2014) and D-loops (Pepe and West, 2014b) and is responsible for the restart of stalled forks in S phase (Gao et al., 2003; Pepe and West, 2014a). When replication forks are stalled in S phase, MUS81–EME2 complex is recruited to cleave the stalled replication forks and induces transient DNA double-strand break (DSB) formation (Gao et al., 2003; Pepe and West, 2014a). The cleavage of MUS81–EME2 restarts the stalled replication forks and promotes replication recovery (Hanada et al., 2007) via break-induced replication (BIR) pathway (Kramara et al., 2018).

### MUS81–EME1 Promotes Faithful Chromosome Disjunction in M Phase

#### MUS81–EME1 Complex Triggers Common Fragile Site Expression

Common fragile sites (CFSs) are difficult-to-replicate foci that preferentially form breaks in chromosomes under replication stress and frequently rearrange in tumor cells (Durkin and Glover, 2007). We revealed that MUS81–EME1 complex promotes expression of CFSs and faithful disjunction of sister chromosomes in human cells (Naim et al., 2013; Ying et al., 2013). Under replication inhibitors, CFSs remain underreplicated at the end of S phase, are prone to form replication intermediates, and then give rise to sister-chromatid bridging in M phase, which contributes to chromosomal instability and oncogenesis (Chan et al., 2009). Nonetheless, MUS81–EME1 complex is phosphorylated and collected to underreplicated CFS loci in early mitosis. RECQ5 dismantles RAD51 from the single-strand DNA (ssDNA), which promotes the cleavage of MUS81–EME1 on stalled replication forks (Di Marco et al., 2017). MUS81–EME1 then cleaves the intertwined DNA strands, manifesting as breaks observed at CFSs (Naim et al., 2013; Ying et al., 2013). Ultimately, we identified that POLD3-dependent DNA synthesis, promoted by MUS81 cleavage, repairs the expressed CFSs and boosts faithful sister chromatid disjunction (Minocherhomji et al., 2015).

#### SLX–MUS Complex Contributes to Holliday Junction Resolution

HJs are cruciform-shaped chromosome junctions that arise temporarily during HR, and their resolution is critical for chromosomal disjunction and genome maintenance (West, 2009). There are three major HJ-resolving pathways in human cells, one of which involves synthetic lethal of unknown function protein 1 (SLX1)–SLX4–MUS81–EME1 (SLX–MUS) complex, a backup for Bloom syndrome protein (BLM)–TopoisomeraseIIIa–RecQ-mediated genome instability protein 1 (RMI1)–RMI2 (BTRR) pathway (Fekairi et al., 2009; Wyatt et al., 2013; Sarbajna et al., 2014). Cells, lacking SLX–MUS complex-associated proteins, show defective chromosome morphology and reduced survival (Wyatt et al., 2013; Sarbajna et al., 2014). After SLX4 and EME1 are phosphorylated by cyclin-dependent kinase, MUS81–MEM1 and SLX1–SLX4 associate and combine into a stable SLX–MUS complex at the G2/M phase (Wyatt et al., 2013). At that point, SLX–MUS complex triggers bilateral cleavage of intact HJs by a coordinated nicking and counternicking mechanism (Wyatt et al., 2013). Interestingly, MUS81–EME1 cleaves intact HJs inefficiently (Ciccia et al., 2003), and SLX1–SLX4 introduces nicks into intact HJs optionally. However, SLX–MUS complex mobilizes cleavage activity of both SLX1–SLX4 and MUS81–EME1 and exhibits a more orchestrated reaction for efficient HJs resolution (Wyatt et al., 2013).

## Relevance Between MUS81 and Cancer

### Involvement of MUS81 in Tumor Suppression

As defects in DNA damage repair are frequently interrelated with high predisposition to cancer, research has been carried out to demonstrate whether MUS81 suppresses tumors *in vivo*. Mus81-deficient mice are born at expected Mendelian frequencies (McPherson et al., 2004), indicating a non-essential role of MUS81 in murine meiotic recombination and oncogenesis. However, MUS81 deficiency, even MUS81 insufficiency, leads to a dramatic susceptibility to cancers, especially lymphomas in mice through the first year of life (McPherson et al., 2004). These tumors show a high frequency of DNA aneuploid via cytogenetic analysis (McPherson et al., 2004). Strikingly, concomitant deficiency of MUS81 and P53 leads to an extremely high frequency in sarcoma development in mice, indicating the collaboration of MUS81 and P53 in tumor suppression (Pamidi et al., 2007). There is, however, a different voice suggesting that murine MUS81 is unnecessary for tumor suppression, based on evidence that no increased sign of tumors was detected in MUS81-deficient mice during a 15-month monitoring (Dendouga et al., 2005). The reason for the discrepant performance of murine MUS81 in tumor predisposition is still unclear. Thus, determining the role of MUS81 in tumor suppression requires further studies. Nonetheless, a growing number of evidence prove that MUS81 plays a dominant role in some specific gene-mutated cancer cells.

### MUS81 Is Essential in BRCA2-Deficient Cancer Cells

**Figure 1:**
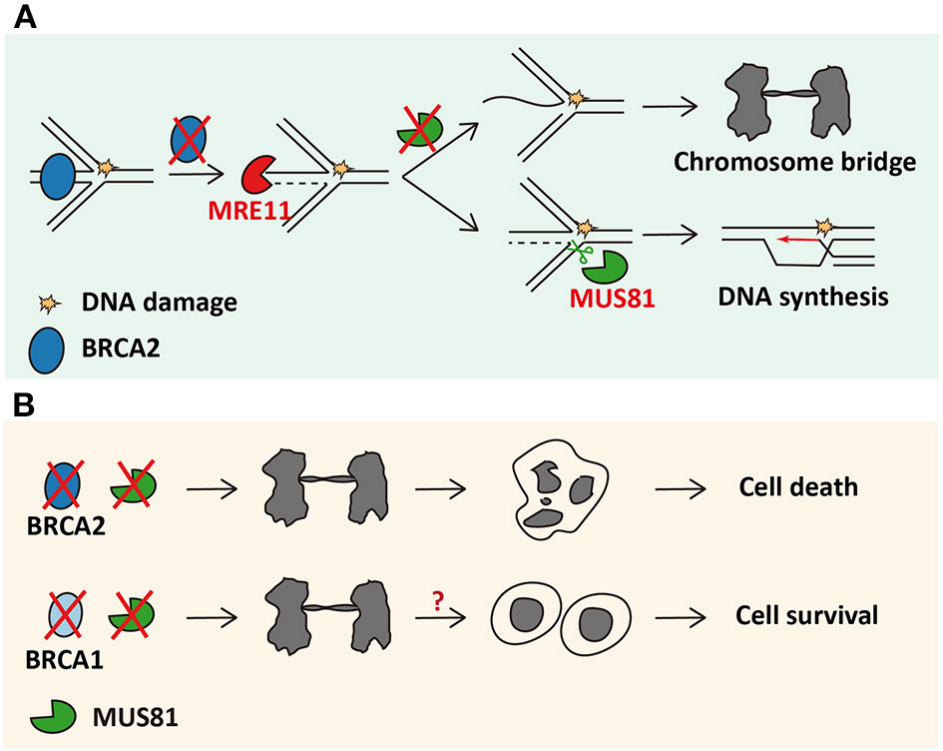
MUS81 sustains survival of BRCA2-deficient cancer cells through rescuing reversed forks. **(A)** The mechanism of MUS81 resolving chromosomal interlinks in cells lacking BRCA2. **(B)** MUS81 is dispensable in BRCA1-deficient cells.

Inheritance of *BRCA2* mutations is responsible for predisposing humans to breast cancer (Ford et al., 1998). Primary human cells with *BRCA2* deletion accumulate spontaneous DNA damage and go toward senescence and apoptosis (Carlos et al., 2013). Mice with homozygous *BRCA2* mutation show embryonic lethality (Jonkers et al., 2001). However, BRCA2-deficient cancer cells can survive in virtue of high tolerance of endogenous DNA damage (Pardo et al., 2020). Dual loss of BRCA2 and MUS81 results in obvious cancer cell death (Lai et al., 2017), indicating a pivotal role of MUS81 in *BRCA2*-mutated cancer cells (Figure 1A). Replication forks stall when they encounter DNA lesions upon drug treatment, and RAD51 promotes reversed fork formation (Zellweger et al., 2015; Lemaçon et al., 2017). Subsequently, BRCA2 is recruited to protect the regressed arms of nascent DNA strands at stalled forks by stabilizing RAD51 filaments from nucleolytic degradation (Schlacher et al., 2011; Lemaçon et al., 2017; Mijic et al., 2017). In the lack of BRCA2, MRE11, initiated by CtIP, targets unprotected reversed forks and starts fork resection by its endonuclease activity (Lemaçon et al., 2017). MRE11 resection leads to the ssDNA flap formation in reversed forks (Lemaçon et al., 2017). MUS81 cleaves these resected regressed forks and leads to transient DSB accumulation (Lemaçon et al., 2017). Finally, POLD3-dependent DNA synthesis repairs DSBs and restarts the MUS81-cleaved forks (Lemaçon et al., 2017). Conversely, in cancer cells with dual loss of BRCA2 and MUS81, resected forks cannot be restarted, which is supported by the observation that frequency of reversed forks with ssDNA is increased dramatically (Lemaçon et al., 2017).

Given that BRCA2 is best known for its function in HR repair, whether the synthetic lethality between MUS81 and BRCA2 can be stretched to other proteins involved in HR pathway, like BRCA1, was further explored. Surprisingly, BRCA1, RAD51, or RAD51C depletion, unlike BRCA2 depletion, cannot lead to synthetic lethality upon concomitant loss of MUS81 in cancer cells (Lai et al., 2017). BRCA1 functions upstream of BRCA2 in a common HR pathway (Roy et al., 2011) and is capable of protecting the reversed forks as BRCA2 does (Lemaçon et al., 2017). Nevertheless, MUS81 and POLD3 foci do not accumulate in BRCA1-insufficient cancer cells (Lemaçon et al., 2017). It would be interesting to uncover the mechanism by which MUS81 insufficiency leads to such different phenotypes between BRCA1- and BRCA2-deficient cancer cells (Figure 1).

### MUS81 Induces Chromosome Shattering in WRN-Deficient MSI Cancer Cells

**Figure 2:**
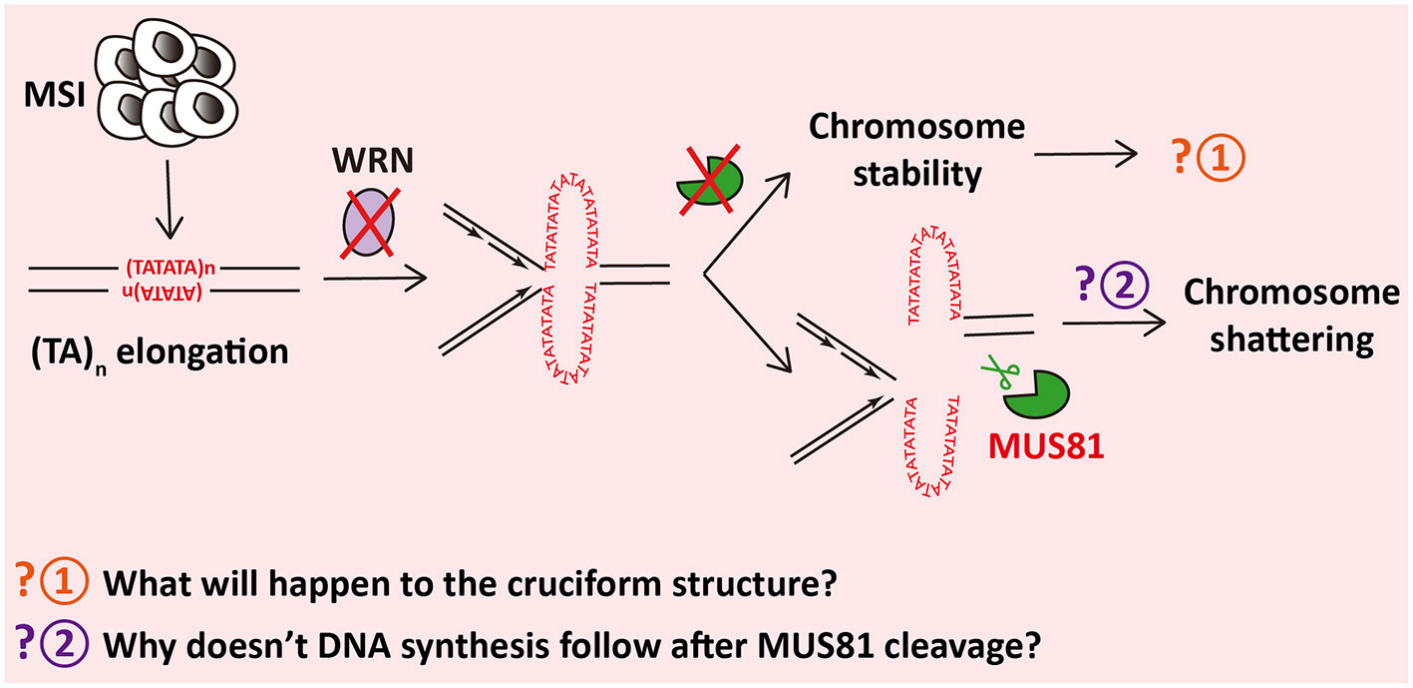
MUS81 shatters chromosomes in WRN-insufficient microsatellite instability (MSI) cells by cleaving (TA)_n_-formed cruciform structures.

MSI is characterized by a hypermutable state of nucleotide repeat regions, which is promoted by defects in DNA mismatch repair (MMR) (Kim et al., 2013). MSI facilitates occurrence of multiple cancers (Pal et al., 2008; Kim et al., 2013). Considering the vulnerabilities of MSI, WRN is identified as an essential gene in MSI cells (Chan et al., 2019). WRN depletion causes DSBs and chromosome shattering (van Wietmarschen et al., 2020) and reduces the cell viability (Chan et al., 2019) in MSI cells but neither in microsatellite stable (MSS) cancer cells nor in primary human cells. However, MUS81 exhaustion before WRN depletion notably cuts down the chromosome shattering and DSBs formation at TA repeats (van Wietmarschen et al., 2020), indicating that MUS81 cleavage contributes to apoptosis of MSI cells with WRN deficiency (Figure 2).

In MSI cells, TA repeats, which are highly unstable, encounter and accumulate large-scale expansions for some reason (van Wietmarschen et al., 2020). Cruciform structures form at the expanded TA repeats (Inagaki et al., 2009), which are tended to stall replication forks. WRN is then recruited and unwinds the secondary structures in virtue of its helicase activity rather than its exonuclease activity, which allows restart of the stalled replication forks (Chan et al., 2019), whereas, in the absence of WRN, cruciform structures of TA repeats cannot be unfolded. Instead, MUS81–EME1 cleaves the cruciform structures and causes DSBs. Interestingly, the cleavage sites are exactly adjacent to the border of expanded TA repeats (van Wietmarschen et al., 2020). Furthermore, cleavage of MUS81 and accumulation of DSBs lead to massive chromosome shattering. Finally, WRN-deficient MSI cells ends in cell cycle arrest and apoptosis (van Wietmarschen et al., 2020).

A question, which remains to be answered, will be of considerable interest. Why does MUS81 cleavage in TA repeats lead to chromosome shattering rather than triggering POLD3-dependent DNA synthesis for DNA repair? It has been found that these TA repeats cannot be amplified *in vitro* and have extremely low sequencing depth (van Wietmarschen et al., 2020), indicating that secondary structures formed at TA repeats might restrain polymerase extension and inhibit DNA synthesis.

## Discussion

DDR pathways play crucial roles in genomic stability, and compromised DDR pathways are common in multiple tumors. Recent studies have unveiled the link between DDR deficiency and innate/adaptive immunity against tumor cells (Barber, 2015; Reisländer et al., 2020). Cyclic GMP–AMP synthase (cGAS) detects the cytosolic DNA caused by DDR deficiency in cancers and activates stimulator of interferon genes (STING), which triggers interferons (IFNs) signaling and antitumor immunity (Chen et al., 2016). DNA mismatch repair (MMR) inactivation-induced neoantigens in cancers boost adaptive immunity as well, which is independent of the cGAS–STING pathway (Germano et al., 2017). Therefore, targeting DDR pathways holds therapeutic potential against cancers.

DDR-directed therapies have been introduced in clinical trials recently (Pilié et al., 2019). However, chromosomal instability (CIN) is the dominant drawback of this strategy, which conversely underpins evolution and growth of tumor cells (Bakhoum and Cantley, 2018; Calzetta et al., 2020). Thus, knowledge of the interactions among multiple DDR pathways and understanding the discrepant functions of DDR-relevant proteins in various cancers contribute to efficient cancer therapy and needs to be further elucidated.

As this review demonstrates, human MUS81 is closely related to cancers but results in totally different fates of cancers bearing distinct mutated genes, indicating that the interactions between MUS81 and other DDR proteins master the cancer cell fate and MUS81 is a potential and intriguing target in specific cancer therapies. A further understanding of the precise mechanisms of MUS81 and the interactions among DDR pathways in discrepant cancers may pave the way toward improved cancer therapeutic strategies.

## Author Contributions

SC wrote the manuscript. XG, MS, ZH, CZ, and SY provided guidance and supervised the final version of the manuscript. All authors contributed to the article and approved the submitted version.

## Conflict of Interest

The authors declare that the research was conducted in the absence of any commercial or financial relationships that could be construed as a potential conflict of interest.
